# Spatio-Temporal-Based Identification of Aggressive Behavior in Group Sheep

**DOI:** 10.3390/ani13162636

**Published:** 2023-08-15

**Authors:** Yalei Xu, Jing Nie, Honglei Cen, Baoqin Wen, Shuangyin Liu, Jingbin Li, Jianbing Ge, Longhui Yu, Yuhai Pu, Kangle Song, Zichen Liu, Qiang Cai

**Affiliations:** 1 College of Mechanical and Electrical Engineering, Shihezi University, Shihezi 832003, Chinaliuzichen@stu.shzu.edu.cn (Z.L.); caiqiang@stu.shzu.edu.cn (Q.C.); 2 Xinjiang Production and Construction Corps Key Laboratory of Modern Agricultural Machinery, Shihezi 832003, China; 3 Industrial Technology Research Institute of Xinjiang Production and Construction Corps, Shihezi 832000, China; 4 College of Information Science and Technology, Zhongkai University of Agriculture and Engineering, Guangzhou 510225, China

**Keywords:** aggression recognition, long short-term memory, YOLOv5, deep learning

## Abstract

**Simple Summary:**

Artificial intelligence technology increases the level of awareness in sheep farming. The aggressive behavior of sheep is related to mortality, feed provisioning, and flock density, and can impact sheep welfare and business benefits in precision animal husbandry. Currently, animal behavior is mainly observed manually, leading to increased labor costs. Contact sensor methods not only increase breeding costs but also induce stress reactions in sheep. The non-contact computer vision method avoids the above problems. Therefore, we propose a deep learning model that combines machine vision and time series analysis. This model aims to meet the requirement of timely and accurate detection of aggressive behavior in large-scale sheep farming.

**Abstract:**

In order to solve the problems of low efficiency and subjectivity of manual observation in the process of group-sheep-aggression detection, we propose a video streaming-based model for detecting aggressive behavior in group sheep. In the experiment, we collected videos of the sheep’s daily routine and videos of the aggressive behavior of sheep in the sheep pen. Using the open-source software LabelImg, we labeled the data with bounding boxes. Firstly, the YOLOv5 detects all sheep in each frame of the video and outputs the coordinates information. Secondly, we sort the sheep’s coordinates using a sheep tracking heuristic proposed in this paper. Finally, the sorted data are fed into an LSTM framework to predict the occurrence of aggression. To optimize the model’s parameters, we analyze the confidence, batch size and skipping frame. The best-performing model from our experiments has 93.38% Precision and 91.86% Recall. Additionally, we compare our video streaming-based model with image-based models for detecting aggression in group sheep. In sheep aggression, the video stream detection model can solve the false detection phenomenon caused by head impact feature occlusion of aggressive sheep in the image detection model.

## 1. Introduction

In recent years, the farming scale of grazing animals, including sheep, pigs, cattle, and others, has been expanding due to the increasing demand for meat and dairy products [1]. The sheep farming industry has been moving towards a large-scale farming model in order to meet the growing demand and reduce production costs. The welfare of grazing animals under the large-scale farming model has gained significant attention [2,3,4]. Sheep, being one of the major grazing animals, their aggressive behavior can be reflective of their welfare level in many ways. Both adding high group density within the sheep pen and feed allocation ratios can affect the frequency of aggression of sheep [5,6,7]. Detection of sheep aggression can help farmers to assess the maximum capacity within the sheep pen and adjust feed ratios in a timely manner. Meanwhile, it alerts the farmer to isolate aggressive sheep, reducing the amount of damage caused to the group sheep. At present, sheep behavior detection is mainly based on manual observation. However, manual observation is inefficient and subjective. Therefore, it is necessary to establish an automatic sheep behavior detection model to improve farming efficiency [8,9].

The present automatic animal behavior detection is primarily divided into contact and non-contact methods. Contact methods requires the animal to wear a sensor. The sensors mainly collect the animal’s motion information, audio information, and chemical information as data, and use machine learning classification algorithms to achieve animal behavior classification. Alvarenga et al. outfitted a three-axis accelerometer on the sheep to obtain the movement information of the sheep, and then used a decision tree algorithm to identify the sheep’s grazing, lying, running, standing, and walking [10]. The different positions of the sensors on the sheep can also affect the detection accuracy. Barwick et al. discovered that the ear-deployed accelerometer had the highest accuracy when the sensors were deployed on the ear, neck, and legs of sheep [11]. Shen et al. designed a method to identify the feeding, ruminating and other behavior of cows based on three-axis acceleration sensor, and then performed comparative analysis of three machine learning algorithms (k-nearest neighbor, support vector machine and probabilistic neural network) [12]. While contact animal behavior detection methods meet accuracy and data processing speed, they cannot be practical for large-scale sheep breeding due to the increased cost associated with outfitting each sheep with sensors. Moreover, the sensors necessitate regular manual battery replacement, which is not suitable for long-term group animal behavior detection [9].

Non-contact methods primarily rely on image processing and machine learning techniques. The process begins by capturing videos of animal behavior, segmenting the target animal to obtain its movement information, and then employing a machine learning classification algorithm to recognize specific behaviors. Viazzi et al. extracted the average intensity and occupation index of sports from the motion history image of pigs, applying linear discriminant analysis to identify aggressive behavior [13]. Gronskyte et al. proposed an improved optical flow method to detect tripping and trampling behavior in pigs [14]. Guzhva et al. extracted geometric features of cows from each frame of the video and utilized the support vector machine to identify social behaviors such as head pressing and body pushing [15]. Chen et al. located aggressive pigs by utilizing a connected area and adhesion index, and then identifying aggressive behavior by extracting acceleration features [16]. Chen et al. subsequently investigated the identification of pig aggression using other motion features [17,18]. Image-processing techniques can effectively segment animals in simple farming environments, so that animal motion features can be further extracted. However, the complexity of the environment in a large-scale open-air breeding model increases the difficulty of extracting the target animals.

With the development of deep learning, artificial intelligence techniques have found extensive applications in agriculture. These applications include crop pest, disease detection, seed quality detection, animal behavior detection, and agricultural data prediction [19,20,21,22,23]. The mainstream methods for animal behavior detection use single-stage detection models such as YOLO (You Only Look Once) [24,25,26,27,28,29] and SSD (Single Shot MultiBox Detector) [30], as well as two-stage detection models such as Faster R-CNN [31]. Thenmozhi et al. compared the performance of SSD model and YOLOv3 model to identify aggression behavior and non-aggression behavior in sheep [32]. Joo et al. and J. Wang et al. used YOLO to recognize fight, stand, feed, drink behaviors in chickens [33,34]. Yu et al. proposed an enhanced YOLOv3 model that combines the EfficientNet-B0 lightweight network and SENet attention mechanism. This model improved the detection speed and accuracy in identifying ewe estrus behavior [35]. Zheng. used Faster R-CNN to identify sow standing, sitting, sternal recumbency, ventral recumbency and lateral recumbency behaviors [36]. In the above methods, static images are used as detection objects. However, animal behavior is continuous, and static image detection ignores the animal’s motion characteristics. Using video streams as the detection objects can preserve the animal’s motion characteristics. Chen et al. and D. Liu et al. combined the SSD model and LSTM (Long short-term memory) model to identify aggressive behavior and tail-biting behavior of pigs [2,37]. The vector was fed into the LSTM model to identify abnormal behavior in pigs. Despite the implementation of video stream detection, the SSD model convolves the image into a one-dimensional vector, which lower the detection speed of the network. Furthermore, these one-dimensional vectors consistently have a length of around 2500. But less related to sheep aggression, which leads to redundant information in the LSTM model.

Aiming at the shortcomings of the image detection model that lacks the motion features of sheep and the video detection model is slow and data redundant. We propose a behavior-detection model of the spatio-temporal-based motion-information extraction of group-sheep aggression, YOLOv5-LSTM. The movement of the sheep was used to predict whether aggression was occurring. In this experiment, we first collect the videos of the sheep pen and edit the video segment where aggression behavior occurs. We then utilize a video keyframe decomposition technique to obtain images from the videos, obtaining both video and image data of aggression behavior. Additionally, we decompose videos of sheep pens from another time period into frames to obtain sheep-detection images. We use open-source software LabelImg to manually label the images with obvious aggression behavior and sheep-detection images with bounding boxes. A sheep-detection image dataset, group-aggression-behavior video dataset and group-aggression-behavior image dataset are obtained.

The model first detects all sheep in each frame of the video by YOLOv5 model and extracts their coordinate information. Secondly, we maintain a constant order of coordinate information output from the sheep in each frame through our proposed sheep-tracking heuristic. Finally, the coordinate information of all sheep in each frame is fed into the LSTM model to predict whether aggression occurs. We compare and analyze the confidence, batch size and frame skipping in the YOLOv5-LSTM model, and compare the experimental optimal YOLOv5-LSTM video-detection model with the YOLOv5 image-detection model to analyze the advantages and disadvantages of the two models.

In summary, this paper makes the following contributions:

(1) A combined YOLOv5 and LSTM model is proposed for detecting aggression in group sheep;
(2) The YOLOv5-LSTM model is compared and analyzed to optimize its performance;
(3) We compare the advantages and disadvantages between the video-based group-sheep-aggression-detection model and the image-based group-sheep-aggression-detection model;
(4) Establishing image datasets and video datasets for group-sheep-aggression, and sheep-detection datasets.

The second section describes the establishment of the dataset and the construction of the YOLOv5-LSTM model. The third section analytes the YOLOv5-LSTM model and compares it with the YOLOv5 model. The fourth section concludes.

## 2. Materials and Methods

### 2.1. Dataset Collection

**Figure 1:**
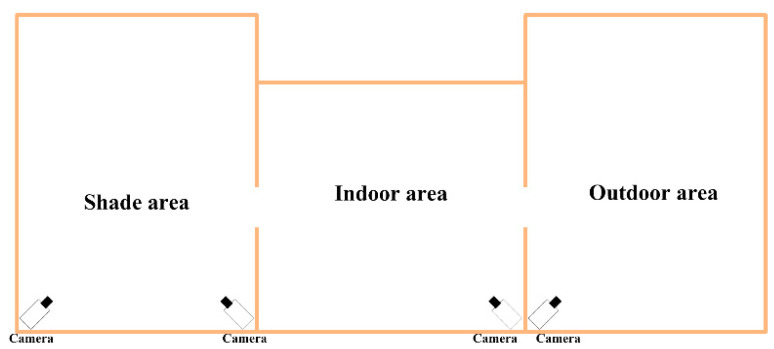
Structure diagram of breeding area and camera layout.

The experimental data were collected from the Xinao animal husbandry mutton sheep breeding base in Lanzhou Bay Town, Manas County, Xinjiang Uygur Autonomous Region. A selected portion of the breeding area was monitored for the study. Each breeding zone consists of three parts: indoor area, shade area and outdoor area. The layout position is shown in Figure 1. The surveillance camera used for monitoring is Hikvision DS-2CD2346FWD-IS Dome Network Camera, resolution 2560 pixels × 1440 pixels, FPS 25 f/s.

Three datasets were prepared for this experiment: sheep-detection dataset, group-sheep-aggression video dataset and group-sheep-aggression image dataset. The YOLOv5-LSTM model was trained on the sheep-detection dataset and the group-sheep-aggression video dataset, while group-sheep-aggression image dataset was trained with the YOLOv5 model. In this study, we defined head-to-head and head-to-body collisions between two sheep accompanied by strong locomotor characteristics as aggressive behaviors of sheep. Behaviors that did not satisfy these characteristics were defined as non-aggressive behaviors, such as standing, prone, walking and running.

#### 2.1.1. The Sheep-Detection Dataset

The first dataset is the sheep-detection dataset, with 321 images of sheep under four cameras randomly collected. To ensure the accuracy of sheep detection in different environments, images containing severe mutual occlusion accounted for 20% (the number of video sheep reached 20 or more), and images at dusk and night accounted for 5%. The images were also horizontally mirrored, vertically mirrored and diagonally mirrored to increase the robustness and diversity of the data. A total of 1284 images were obtained after data augmentation. The images were manually annotated with the label “sheep” to build a sheep-detection dataset. The dataset was divided 8:2:2 with training, validation and test datasets.

#### 2.1.2. The Group-Sheep-Aggression Video Dataset

The second dataset is a group-sheep-aggression video dataset, which recorded starting at the aggressive sheep’s violent movement behavior until the aggressive sheep’s movement calmed down. Sheep aggression is usually a repeated behavior. The aggressive sheep’s behaviors, such as mutual explorations before head impacts, touching and squeezing without obvious injuries, were used as non-aggression videos. Additionally, non-aggression videos were selected in part as daily behaviors and in part as pre-aggression videos to increase the distinction between aggressive and non-aggressive behaviors in training. A total of 178 aggression videos and 129 non-aggression videos were obtained from the group of sheep. To increase data diversity, the data were horizontally, vertically and diagonally mirrored. Furthermore, the video data was randomly divided into training and test sets in an 8:2 ratio [37]. Through video observation, the minimum time of the aggressive behavior of the sheep was 1 s. Therefore, the video data were further divided into 1 s sub-videos. The training set of the aggressive and non-aggressive class consisted of 2126 and 2175 examples, respectively. The test set of aggressive and non-aggressive class consisted of 430 and 455 examples, respectively.

#### 2.1.3. The Group-Sheep-Aggression Image Dataset

The third dataset is the group aggression image dataset. The group-sheep-aggression videos were decomposed into frames. The images with obvious aggression behavior were selected, such as head-to-head impacts and head-to-body impacts between aggressive sheep, accompanied by the twisting of the sheep’s body following the impact. A total of 3218 sheep-aggression images were obtained, and each image was manually labeled as “aggression”. This established the sheep aggression dataset. The dataset was then divided into training, validation and test in a ratio of 6:2:2 [38].

### 2.2. YOLOv5-LSTM Model Construction

#### 2.2.1. YOLOv5 Introduction

During recent years, the YOLO series network models have gained popularity in the field of image object detection. This is due to their fast detection speed, high recognition accuracy, and ease of deployment on mobile and embedded devices, thus meeting the practical detection requirements.

**Figure 2:**
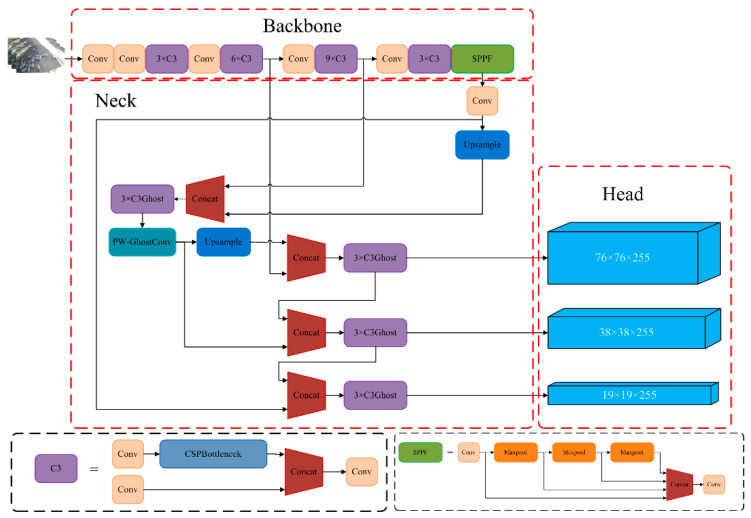
YOLOv5 network structure diagram.

YOLOv5 model consists of three main components: Backbone, Neck and Head, as shown in Figure 2. The Backbone incorporates an enhanced version of the CSPDarkNet53 and SPPF (Spatial Pyramid Pooling). The improved CSPDarkNet53 network is used to extract image features, SPPF fuses local and global feature information. The Neck primarily consists of FPN (top-down feature pyramid) and PAN (top-up feature pyramid), which combine feature maps from network layers of different depths to enrich semantic information. Lastly, the head is constructed of three Detect layers, which can predict results.

The YOLOv5 model divides the input image into S × S grids. Each grid is randomly assigned three different sizes of anchor and predicts the class, confidence, and coordinate information $\left[\mathrm{obj},\;x,\;y,\;w,\;h\right]$ for the objects in each grid. Where obj denotes the confidence of the object in the grid, $\left(x,\;y,\;w,\;h\right)$ denote the coordinates of the prediction box’s center $\left(x,\;y\right)$ and its width and length $\left(w,\;h\right)$. Therefore, the YOLOv5 loss function consists of three components: position loss, confidence loss and classification loss. Non-maximum suppression is utilized to prevent multiple predicted boxes from being generated for the same object.

The effectiveness of the object detection model needs to be evaluated for practical production use. We use the P (Precision), the R (Recall) and F1 as the model for accuracy evaluation, and the calculation formula is shown in (1).

(1) $$\left\{\begin{array}{c} P=\frac{TP}{TP+FP}\\ R=\frac{TP}{TP+FN}\\ F1=2\times \frac{\mathrm{PR}}{P+R} \end{array}\right.$$

where TP (True Positives) denotes the number of positive samples identified in the positive samples, FP (False Positives) denotes the number of positive samples identified in negative samples, FN (True Negatives) denotes the number of positive samples identified as negative, and F1 is the comprehensive evaluation index of P and R.

#### 2.2.2. LSTM Introduction

Long short-term memory neural networks (LSTM) are a variant of recurrent neural networks (RNN) [39]. They are extensively employed in prediction models for time series data. However, compared with RNN, LSTM can effectively capture the contextual information in long sequences and effectively address the problem of gradient disappearance or exploding. As a result, the LSTM model is chosen to extract the features in the temporal dimension.

**Figure 3:**
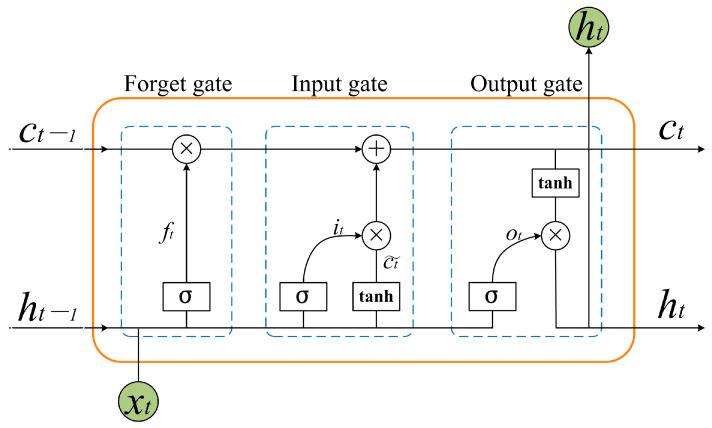
Schematic diagram of LSTM network structure.

An LSTM neuron comprises three gate structures: the forgetting gate ($f_{t}$), the input gate ($i_{t}$) and the output gate ($O_{t}$), as shown in Figure 3. Let the moment be denoted as t. The output of the neuron at moment t−1 is $h_{t-1}$, and the input data are $x_{t}$. The $f_{t}$, $i_{t}$, $O_{t}$ and $h_{t}$ are calculated as shown in (2).

(2) $$f_{t}=\sigma \left(W_{f}\left[h_{t-1},x_{t}\right]+b_{f}\right)\;i_{t}=\sigma \left(W_{i}\left[h_{t-1},x_{t}\right]+b_{i}\right)\;O_{t}=\sigma \left(W_{o}\left[h_{t-1},x_{t}\right]+b_{o}\right)\;h_{t}=O_{t}\times \tan h\left(C_{t}\right)$$

where σ denotes the activation function Sigmod(), $W_{*}$ denotes the weight, and $b_{*}$ denotes the bias. The input data comprise the YOLOv5 to detect the coordinate information [x, y, w, h] of each sheep every frame and normalize the data, as shown in Equation (3). The LSTM network extracts the temporal features from the sheep’s coordinate information in each frame, while the fully connected network fuses the temporal features of each frame. Finally, the Sigmod() activation function to predict whether aggression occurs. The training dataset used for this model is labeled as 1 and 0, where 1 indicates aggression in the video, and 0 indicates the non-aggression. As this is a binary classification task, the binary cross-entropy loss function (as shown in Equation (4)) is chosen.

(3) $$x^{\prime }=\frac{x-\min\left(x\right)}{\max\left(x\right)-\min\left(x\right)}$$

(4) $$Loss=-\frac{1}{N}\overset{N}{\underset{i=1}{\sum }}\left[y_{i}\log\left(p_{i}\right)+\left(1-y_{i}\right)\log\left(1-p_{i}\right)\right]$$

where x denotes the input data, $x^{\prime }$ denotes the x-normalized data. N denotes the total number of samples, $y_{i}$ denotes the class to which the i sample belongs, and $p_{i}$ denotes the predicted value of the i sample. In the LSTM model, both P and R are considered evaluation metrics for measuring the accuracy of aggressive behavior recognition.

#### 2.2.3. Sheep Tracking Heuristic

YOLOv5 detects all sheep in each frame and outputs the coordinate information for each sheep. It is crucial to maintain consistency between the coordinates of aggressive behavior and the input units of the LSTM model. The order of outputting the coordinate information of all sheep in each frame follows the sequence in which YOLOv5 detects them, ranked from the highest to the lowest confidence value. During actual detection, the sheep in the video are in motion, resulting in changing confidence values. As a result, the order of the coordinate information for all sheep in each frame constantly changes, making it difficult to correspond to the input units of the LSTM network one by one. Moreover, false detections and missed detections in each frame impact the consistency of the order and length of the input data for the LSTM model.

**Figure 4:**
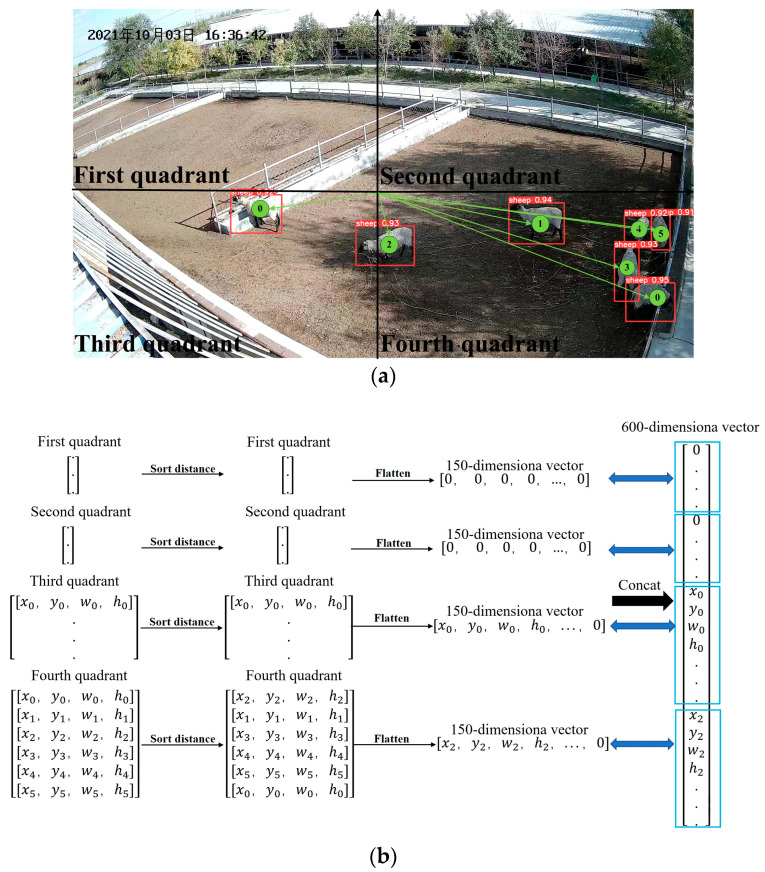
Algorithm process: (**a**) YOLOv5 sheep-detection schematic: The numbers in the green circles denote the order of the output after YOLOv5 detection in corresponding quadrant; (**b**) process chart of sheep tracking heuristic. The Chinese characters in the upper left corner of the image are the date of the video recording, in order of year, month, and day.

We watched the sheep aggression video and found that the coordinate information for each sheep changed relatively little compared to the overall length of the image in the 1 s sub-video. Therefore, we can sort the coordinate information for all the sheep in each frame based on the distance from the image origin (0, 0), from smallest to largest. This sorted order is used as the output order for the coordinate information of all sheep in each frame. However, there is still a possibility of inconsistent input data due to false and missed detections. In YOLOv5, the default position for the image origin is set at the upper left corner of the image. From Figure 4, it can be seen that the center of the image is off the lower right region close to the camera. Consequently, the confidence level for sheep in this region is higher, and it is not easy to misdirect phenomena. On the other hand, if a sheep appears in the upper left corner, it is further away from the camera, resulting in a lower confidence level and making successful detection in every frame challenging. This causes inconsistent input data for the LSTM model. To address this issue, the image origin is set at the midpoint of the image, which is the central area of the sheep pen. Sheep will frequently occur aggression in the center of the sheep pen, and sheep characteristics are obvious, so it is not easy to false and missed detection.

Based on the analysis above, we performed the following steps on the input data for the LSTM model. The image was divided into four quadrants based on the Cartesian coordinate system. From our surveillance video observations, the maximum number of sheep that can be accommodated in a sheep pen did not exceed 70 sheep. Therefore, we set the maximum number of sheep in the pen to 150. Each sheep’s coordinate was represented by four coordinates [x, y, w, h], so the length of the input data was 600-dimensional. For each quadrant, we specified a length of 150 dimensions for the input data. Firstly, we sorted the distances from the sheep’s positions to the origin of the image center in each quadrant, in order to determine the input data order for the coordinate information of all the sheep in each quadrant. Then, we added zeros to the input data to satisfy the 150-dimensional length. Finally, the data from the four quadrants were concatenated. The algorithm process is shown in Figure 4. In Figure 4a, the red box represents the prediction box determined by [x, y, w, h]. The number in the upper left corner of the prediction box is the confidence value, and the numbers inside the box denote the output order of YOLOv5 detection boxes in the corresponding quadrant. This algorithm can maintain the coordinate information of the aggressive sheep corresponding to the input units of the LSTM model well.

**Figure 5:**
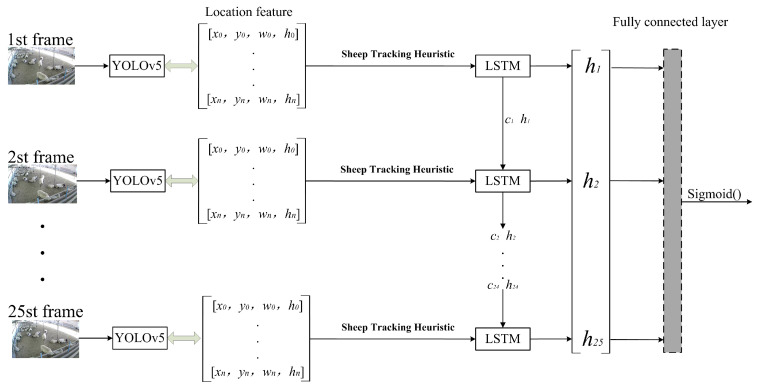
YOLOv5-LSTM model structure.

The YOLOv5-LSTM model is shown in Figure 5. The YOLOv5 model detects all the coordinate information of sheep in each frame and maintains the consistent output order of the sheep using a sheep tracking heuristic. The coordinate information of each sheep frame is then fed into the LSTM model framework. The LSTM model extracts temporal features from the information, distinguishing between aggressive and non-aggressive sheep. The features are fused in by the fully connected network. Finally, the Sigmod() activation function is applied to predict whether aggression occurs in the 1 s sub-video.

## 3. Results and Discussion

### 3.1. Model Analysis

The hardware used for this experiment is shown in Table 1. The YOLOv5 model has an initial learning rate and cycle learning rate of 0.01, a weight decay of 0.005, a batch size of 8, and retains the pre-training weight. The model is trained for 300 epochs using the SGD optimizer. The LSTM model has an initial learning rate of 0.01 and the model is trained for 200 epochs using the Adam optimizer. The batch size of the LSTM model is the length of one sub-video frame number according to Appendix A.

**Figure 6:**
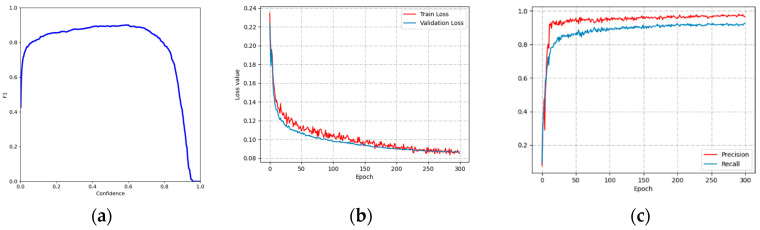
Metric change curve: (**a**) YOLOv5 F1-Confidence; (**b**) YOLOv5 Loss-Epoch; (**c**) YOLOv5 P, R-Epoch.

Figure 6 shows the metrics’ variation as the sheep-detection dataset was trained using YOLOv5. The accuracy of sheep-detection directly impacted the prediction results of the LSTM model. In scenarios with dense groups of sheep, setting a low confidence threshold may lead to numerous false detections, including multiple prediction boxes for a single sheep. Therefore, adjusting the confidence threshold to a higher value can help reduce the occurrence of false detections. Figure 6a shows the F1 and confidence curves for the test set; it can be observed that the F1 value reaches its highest point at a confidence value of 0.6, where it attains a value of 0.90. Therefore, the confidence value for the YOLOv5 sheep-detection model is set at 0.6.

Since some images contained dense sheep, even if the pre-trained weights are retained, it still takes for the loss function to converge during training. In Figure 6b, the YOLOv5 model exhibits a slight oscillation in the training loss function around 0.08 after 250 epochs, while the validation loss function fluctuates around 0.09. Figure 6c shows the P, R curves for the training set, both of which oscillated between 96.5% and 92% over the course of 250 epochs. Due to the dense sheep, there were several missed detections, resulting in a lower value for R compared to P.

**Figure 7:**
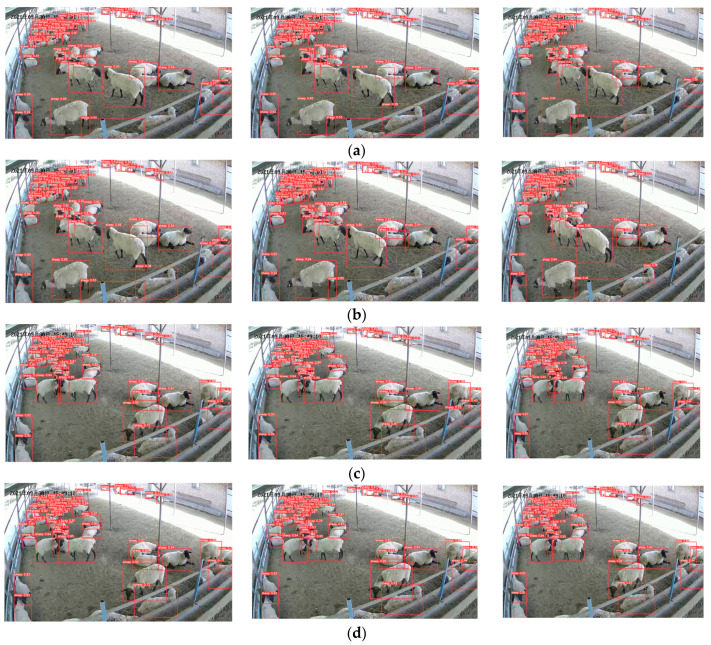
Time series analysis of frame skipping: (**a**) aggressive 1 s 25 frames; (**b**) aggressive 1 s 12 frames; (**c**) non-aggressive 1 s 25 frames; (**d**) non-aggressive 1 s 12 frames. The Chinese characters in the upper left corner of the image are the date of the video recording, in order of year, month, and day.

Skipping frames can effectively reduce the number of sub-video frames, thereby reducing both the training and detection time. Meanwhile, it can enhance the detection of the aggressive-behavior motion features. As shown in Figure 7, the aggressive and non-aggressive-behavior time series analyses were performed by taking each 5 frames without skipping frames (1 s 25 frames) and skipping 1 frame (1 s 12 frames). Figure 7a,b illustrates the time series analysis of sheep movement during aggressive behavior. When skipping 1 frame, the changes in the sheep-movement coordinate information are more pronounced. Additionally, when examining non-aggressive behavior, skipping frames does not significantly change the coordinate information in Figure 7c,d.

**Figure 8:**
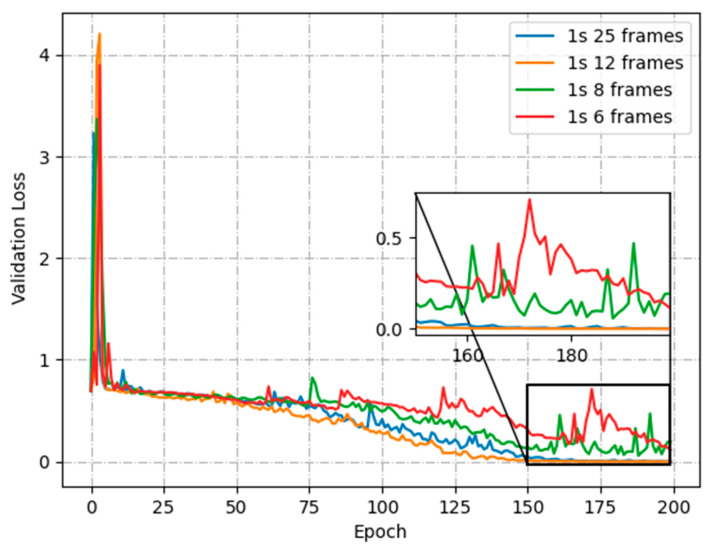
Validation loss for skipping frame.

To further analyze the effect of the different number of skipped frames on the training process, Figure 8 shows the loss function curves with no frame skipping (1 s 25 frames), skipping 1 frame (1 s 12 frames), skipping 2 frames (1 s 8 frames) and skipping 3 frames (1 s 6 frames), respectively. Compared with no-frame skipping, the loss function converges more quickly when skipping 1 frame. It is more difficult to converge while increasing the skipped-frame amplitude. The excessive skipped-frame amplitudes are prone to misclassify non-aggressive activity in sheep videos as aggressive behavior, while also reducing the amount of motion information available for aggressive sheep. By skipping 1 frame, it is possible to save half the YOLOv5 detection time and LSTM model training time.

**Figure 9:**
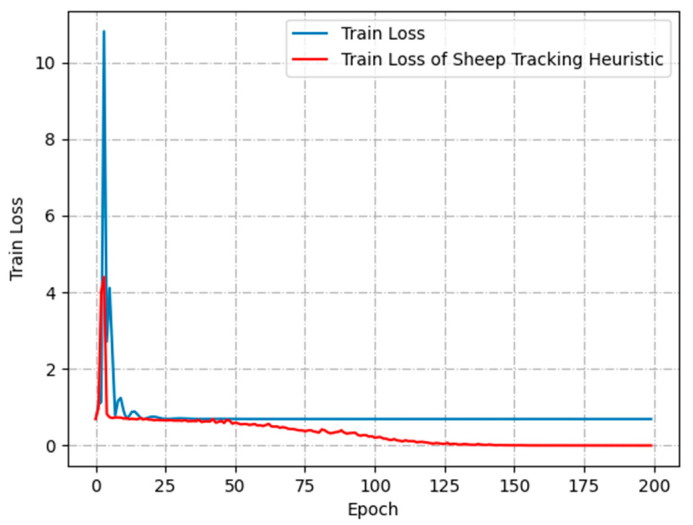
Comparison of training loss functions.

Figure 9 presents the comparison between the loss function of the sheep-tracking heuristic and the no-sheep-tracking heuristic. Without using the algorithm proposed in this paper, the loss function of the LSTM model remains stagnant after 25 epochs and converges around 0.69. However, when using the sheep tracking heuristic, the loss function continues to decrease until it converges near 0. This verifies that the sheep tracking heuristic introduced in this paper ensures that the coordinate information of most sheep in the sub-video corresponds to the input units of the LSTM model.

**Figure 10:**
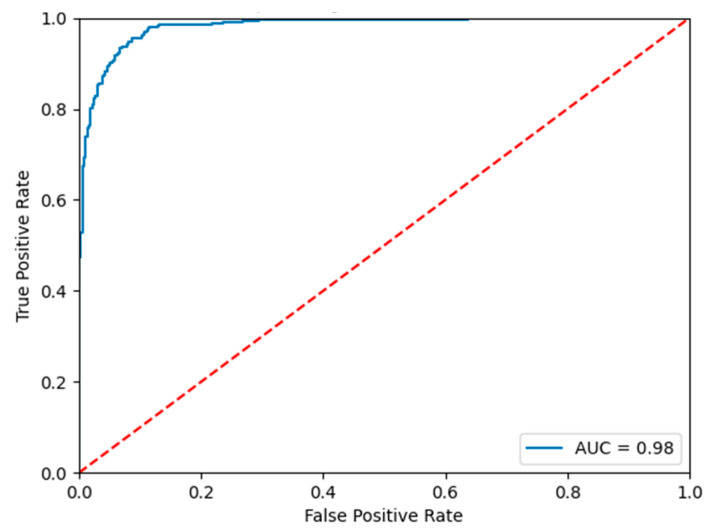
ROC curves of the test set.

Table 2 and Figure 10 show the confusion matrix and ROC curve plots for the test set. From them, we can see that our proposed model has a good classification effect on sheep aggression.

**Figure 11:**
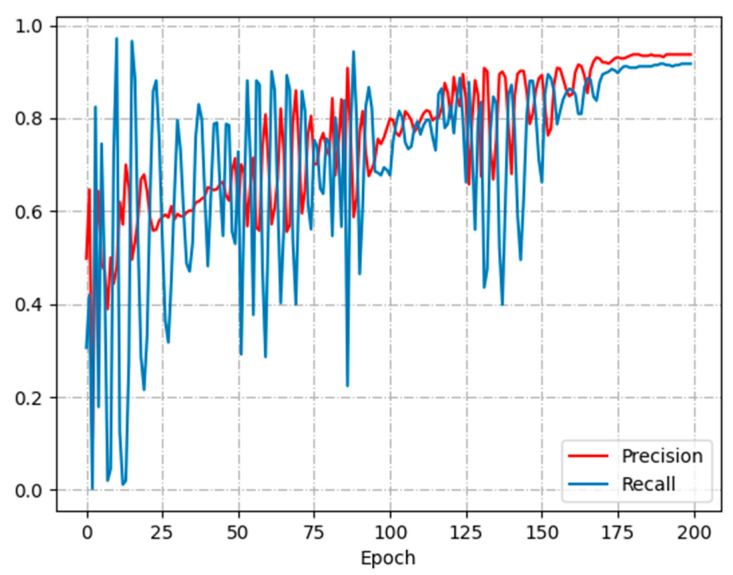
P-R curve.

Figure 11 illustrates the change curves of P and R metrics during the training of the LSTM model. The model changes drastically in P and R values before 150 epochs. After 175 epochs P and R start to converge. The P converges at 0.93 and the R converges at 0.91. The final training model achieves P of 93.33% and R of 91.74% when detecting the test dataset.

**Figure 12:**
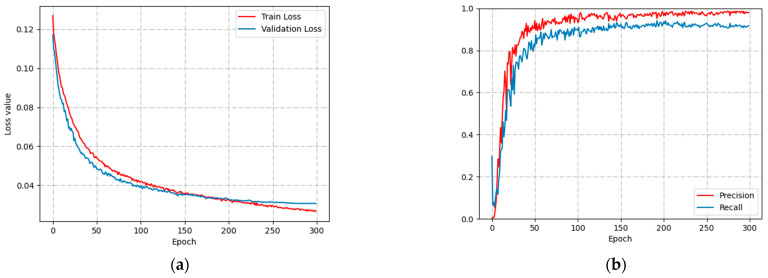
Loss and P-R curves: (**a**) Loss value; (**b**) P-R curve.

Figure 12 shows the variation of loss function and P-R curve of YOLOv5’s image detection model used to identify aggressive behavior in group sheep. Figure 12a shows the loss functions of the training and validation. The training loss function converges at 0.025 and gradually decreases, and the validation loss function converges around 0.03 after 200 epochs. Figure 12b shows the P-R curves, where P stabilizes around 0.95 and R stabilizes around 0.9 after 200 epochs. The final model P and R values of 95.4% and 88.7%, respectively. In a densely populated environment, head impact features of aggressive sheep are easily obscured by non-aggressive sheep, resulting in lower R values than P values.

### 3.2. Analysis of Identification Results

**Figure 13:**
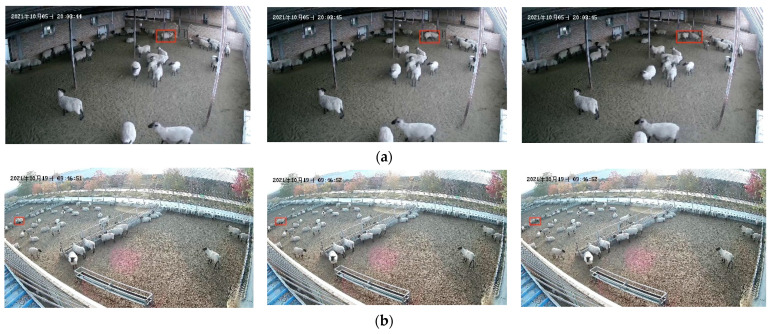
Misidentification of aggressive behavior: (**a**) courtship of sheep; (**b**) Sheep at the edge of the field of vision. The Chinese characters in the upper left corner of the image are the date of the video recording, in order of year, month, and day. The red squares are manually labeled sheep aggression.

The YOLOv5-LSTM model based on video streams is proposed in this paper. It does not make a good judgment when the sheep aggression occurs in the corner of the surveillance field of view and the sheep courtship case. Figure 13 shows several examples of misidentification by the model. Figure 13a shows the courtship behavior of sheep, where several rams chasing ewes. The temporal changes in coordinate information closely resemble aggressive behavior, making it difficult to distinguish between courtship and aggression. Figure 13b shows aggressive sheep at the edge of the visual field. Since the size of the sheep is relatively small, the YOLOv5 model detects these sheep with low confidence. Additionally, the normalization does not distinguish whether the sheep are near or far from the camera.

**Figure 14:**
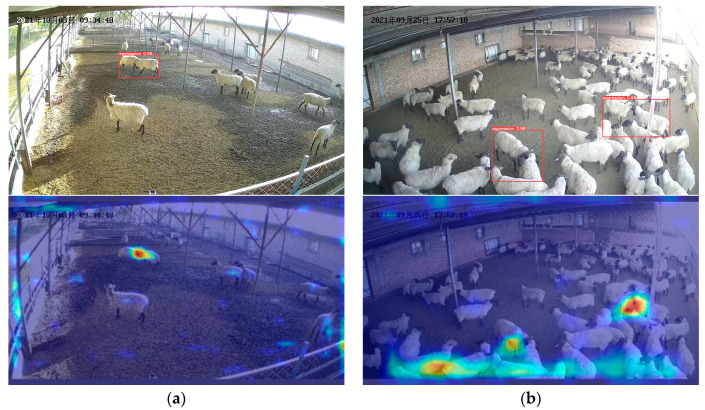
YOLOv5 model detection: (**a**) Single aggression events; (**b**) Intensive aggression events. The Chinese characters in the upper left corner of the image are the date of the video recording, in order of year, month, and day.

Figure 14 illustrates several images and heat maps generated by the YOLOv5 image detection model. The heat map is uniformly selected from the small target detection layer of YOLOv5, where the brighter colors indicate higher model sensitivity at those locations. Figure 14a illustrate the rapid capture of the aggressive character of head impacts at low flock densities. However, when sheep are densely populated, false detection are more likely to occur, as illustrated in Figure 14b.

In a large-scale farming model, the welfare level of animals can be improved by observing their behavior. Therefore, many scholars establish different animal-behavior detection models, and most of the animal-detection models detect static images, neglecting the animals’ movement information. Chen et al. and D. Liu et al. combined the SSD model and LSTM model to detect videos. Although the above problems were solved, the slow detection speed of the SSD model and the redundancy of the input data of the LSTM model remained unsolved. We replaced the SSD model with YOLOv5, which has a faster detection speed. Additionally, the input information of the LSTM model is also changed from the convolved feature maps to the coordinate information of the sheep, which reduces the number of parameters and computation of the LSTM model.

## 4. Conclusions

In order to implement video-based sheep aggression detection, this paper proposes a YOLOv5-LSTM model, which combines the YOLOv5 model and LSTM model. The coordinate information of sheep in each frame is sorted by sheep tracking heuristic. The coordinate information of all sheep in each frame is input into the LSTM model to predict whether aggression occurs. The confidence, batch size and skipping frame were analyzed, and suitable parameters were selected through comparison tests. The final model had a P of 93.38%, R of 91.86% and AUC of 0.98.

In this study, we proposed two methods for detecting aggression in group sheep: video stream-based detection and image-based detection. Video stream-based detection methods incorporate time dimension information to reduce false detections and improve accuracy. However, they are unable to localize aggression events within the video frames. On the other hand, image-based detection methods have the advantage of localizing aggression events, but they are more susceptible to false detections when the density of the group of sheep is high.

Follow-up work can be combined with optical flow methods to achieve the localization of aggression, and also by using advanced object tracking algorithms to achieve the precise localization of each sheep in the video.

## Figures and Tables

**Table 1:** Experimental configuration environment.

Configuration	Parameter
CPU	Inter(R) Core (TM)i7-9700KCPU@3.2GHz
GPU	NVIDIA GeForce RTX 2080
Operating system	Windows 10
Development environment	Pycharm 2021

**Table 2:** The confusion matrix of test set for group sheep aggression.

	Prediction	Positive	Negative
Reference			
Positive		395	35
Negative		28	427

**Figure A1:**
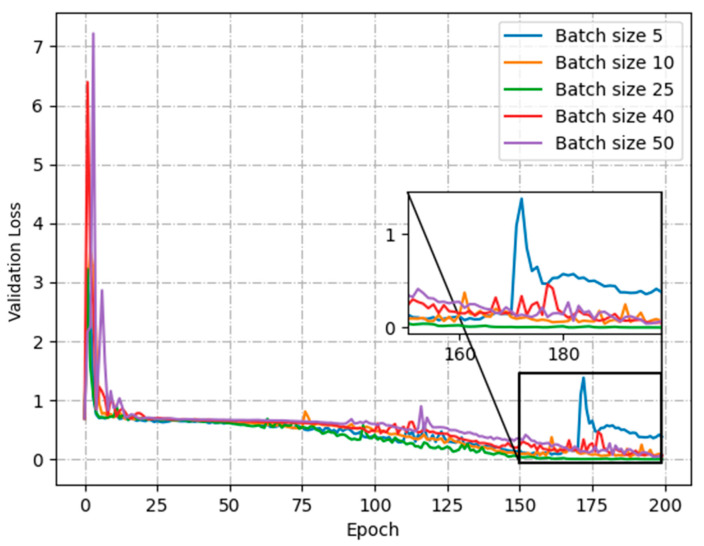
Validation loss function of the LSTM model.

## Data Availability

The data presented in this study are available on request from the cor-responding author. The data are not publicly available due to these data are part of an ongoing study.

## Appendix A

Batch size can affect the training speed and accuracy of the model. We verified the performance of different batch size values during training. We divided 1 s sub-video with a 25-frame criterion and conducted five separate experiments. Figure A1 illustrates the change in validation loss function curves of the LSTM model for batch sizes of 5, 10, 25, 40, and 50. It can be seen that the verification loss function converges fastest only when the batch is 25. When the batch size is less than a sub-video frame numbers, it will destroy the integrity of the sheep-motion information in the sub-video. When the batch size is greater than a sub-video frame numbers, it will mix in the other sub-video sheep motion information and affect the training model. Therefore, in this paper, the batch size is set to the numbers of a sub-video frame.
